# Amyotrophic Lateral Sclerosis (ALS) Type 8: A Narrative Review

**DOI:** 10.7759/cureus.76717

**Published:** 2025-01-01

**Authors:** Billy McBenedict, Wilhelmina N Hauwanga, Uzma Nezam, Aung Ko Oo, Srilatha Eapi, Swetapadma Pradhan, Ngoc B Dang, Phoh Wen Cher, Marco A Orsini, Bruno Lima Pessôa

**Affiliations:** 1 Neurosurgery, Fluminense Federal University, Niterói, BRA; 2 Cardiology, Gaffrée and Guinle University Hospital, Federal University of the State of Rio de Janeiro, Rio de Janeiro, BRA; 3 Biomedical Sciences, Monash University, Melbourne, AUS; 4 Medicine, AMA School Of Medicine, Makati, PHL; 5 Internal Medicine, California Institute of Behavioral Neurosciences and Psychology, Fairfield, USA; 6 Internal Medicine, European University Faculty of Medicine, Tbilisi, GEO; 7 Nursing, College of Health Sciences, VinUniversity, Hanoi, VNM; 8 Orthopaedic Surgery, IMU University, Kuala Lumpur, MYS; 9 Neurology, Federal University of Rio de Janeiro, Rio de Janeiro, BRA

**Keywords:** als type 8 (als8), amyotrophic lateral sclerosis (als), fus mutations, gene therapy, precision medicine, rna-based therapies, stem cell treatments, symptom management, unfolded protein response (upr), vapb gene mutation

## Abstract

Amyotrophic lateral sclerosis type 8 (ALS8) is a rare familial subtype of ALS caused by mutations in the vesicle-associated membrane protein-associated protein B (VAPB) gene, particularly the p.P56S mutation. It is distinguished by slower disease progression and an earlier onset compared to sporadic ALS forms, along with unique clinical features such as severe cramping, fasciculations, postural tremors, and cognitive and behavioral impairments. Although current pharmacological options, such as riluzole, edaravone, and sodium phenylbutyrate/taurursodiol, provide modest benefits, they fail to address the underlying genetic mechanisms of ALS8. Emerging gene therapies, RNA-based interventions, and stem cell approaches hold promise for precision-targeted treatments but face challenges in clinical application. Symptom management strategies, including respiratory, nutritional, and psychological support, are crucial for improving patient outcomes and quality of life. Despite significant progress in understanding the genetic and molecular pathogenesis of ALS8, its rarity, phenotypic variability, and limited clinical data pose challenges to therapeutic advancements. This narrative review highlights current therapeutic strategies, the unique clinical trajectory of ALS8, and potential pathways for innovative, subtype-specific interventions, emphasizing the need for multidisciplinary and targeted approaches to optimize care for this distinct ALS subtype.

## Introduction and background

Amyotrophic lateral sclerosis (ALS) is a severe neurodegenerative disorder characterized by the progressive degeneration of both upper and lower motor neurons within specific regions of the brainstem and spinal cord, such as the cervical, thoracic, or lumbosacral levels. This leads to muscle weakness, paralysis, and eventual respiratory failure [1,2]. The exact cause of ALS remains unknown, and there is no effective cure [3]. Early symptoms typically include fasciculations, muscle cramps, spasticity, and localized muscle weakness in the limbs or neck. As the disease progresses, patients experience dysarthria, dysphagia, muscle atrophy, sialorrhea, dyspnea, and pseudobulbar affect [4,5]. In addition, ALS patients often face negative impacts on their quality of life, such as depression, feelings of hopelessness, anxiety, fatigue, and pain, further compounding the disease burden [6].

The prognosis for ALS is poor, with a median survival time of 2-5 years, although some individuals survive for 10-20 years or longer [7,8]. Chio et al. [3] noted that ALS exhibits a considerable variability in outcome, and its prognostic factors are not yet clearly defined. Approximately 50% of ALS patients die within 30 months of symptom onset, often due to respiratory insufficiency [9]. Risk factors include both lifestyle-related and occupational or environmental factors. Lifestyle factors include smoking, antioxidant intake, physical fitness, body mass index, and physical activity. Occupational and environmental exposures involve risks such as electromagnetic fields, metals, pesticides, β-methylamino-L-alanine, and viral infections [9].

As ALS progresses, patients face profound impacts on their quality of life, including physical limitations, challenges in maintaining weight and nutrition, emotional distress, and social isolation. These factors contribute to depression, hopelessness, anxiety, and fatigue [3,6]. Ultimately, ALS imposes a dual burden of physical and psychological challenges on both patients and their families. Among the various forms of ALS, ALS type 8 (ALS8) stands out as a genetically and clinically distinct subtype, primarily characterized by mutations in the vesicle-associated membrane protein-associated protein B (VAPB) gene, specifically a missense mutation substituting proline for serine at position 56 [10]. ALS8 typically manifests as a slowly progressive familial disorder, predominantly affecting lower motor neurons and presenting with symptoms such as fasciculations, postural tremors, and severe cramping [11]. While ALS8 is distinct from other ALS subtypes, such as ALS1 and ALS2, in its slower progression, some studies suggest it has an earlier onset compared to sporadic forms [11]. The unique genetic basis and clinical features of ALS8 highlight the importance of focusing on this subtype to better understand the disease mechanisms and develop subtype-specific therapeutic interventions [10,12].

Additionally, the potential cognitive and behavioral impairments associated with ALS8 underscore the need for integrated care strategies encompassing both neurological and psychological supports [5]. Current treatments for ALS focus on symptom management and slowing disease progression through pharmacological agents like riluzole, edaravone, PB-TURSO, and Qalsody. However, their efficacy remains modest, and they are often accompanied by side effects, such as liver toxicity or gastrointestinal disturbances [13,14]. 

For ALS8, therapeutic options remain limited, but gene therapy shows promise in modulating VAPB expression and may complement existing pharmacological treatments as research advances [15,16]. Despite advances, most therapies remain palliative, highlighting the need for further exploration of targeted and combination strategies to improve outcomes for ALS8 and other subtypes. This review aims to provide a comprehensive overview of ALS8, emphasizing its unique genetic and clinical features, current therapeutic strategies, and the potential for novel, targeted approaches. By synthesizing available research, we seek to identify the critical gaps in understanding ALS8 and explore potential tailored interventions that could ultimately contribute to improved outcomes for patients with this rare ALS subtype.

## Review

**Figure 1:**
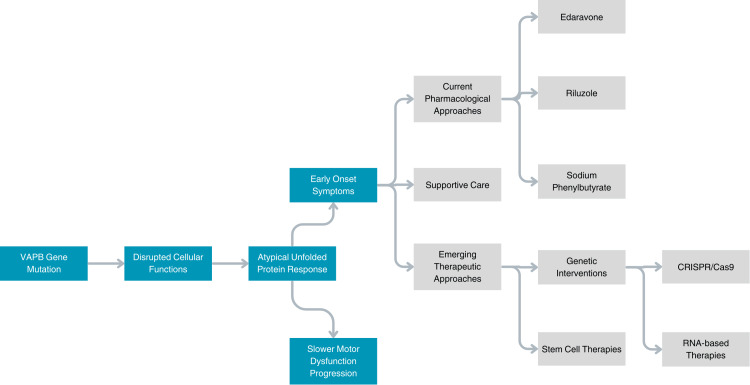
Concept map for ALS8. Image created using information from various references [10-32]. VAPB: vesicle-associated membrane protein-associated protein B, CRISPR: clustered regularly interspaced short palindromic repeats.

ALS8 presents unique challenges and opportunities in therapeutic management due to its distinct genetic underpinnings and clinical trajectory. The interplay between genetic mutations, disrupted cellular pathways, and slower disease progression creates an opportunity for tailored interventions that target its specific pathophysiology. While existing pharmacological treatments provide symptomatic relief, they do not fully address the underlying mechanisms of disease progression. Recent advances in genetic and experimental therapies highlight the potential for precision medicine, offering hope for more effective disease-modifying strategies. Additionally, the integration of supportive care with emerging treatments emphasizes the importance of a multidisciplinary approach to improve both functional outcomes and quality of life for patients with ALS8. This discussion delves into these therapeutic innovations, exploring how they align with the molecular complexities and clinical demands of this rare ALS subtype, as depicted by the concept map in Figure 1. 

Overview of ALS8 and its unique clinical features

ALS8 is a rare familial form of ALS caused by a missense mutation in the VAPB gene [11]. The mutation involves the substitution of proline for serine at position 56 (p.P56S), which disrupts cellular membrane trafficking and endoplasmic reticulum (ER) function [10]. This subtype is clinically notable for its early onset, typically in the 30s or 40s, and its unique symptoms, including fasciculations, postural tremors, and severe cramping, along with early limb weakness and muscle atrophy [5,10]. Unlike most other ALS subtypes, ALS8 features a slower progression of motor dysfunction, allowing patients a longer period of functional independence, though respiratory and bulbar complications eventually emerge (Table 1).

**Table 1:** Comparison of ALS type 8 vs other ALS subtypes. ALS: amyotrophic lateral sclerosis, RNA: ribonucleic acid, FUS: fused in sarcoma, SOD1: superoxide dismutase 1, TARDBP: TAR DNA-binding protein, C9orf72: chromosome 9 open reading frame 72.

Feature	ALS8	Other ALS subtypes	Citation
Onset age	30s-40s (early)	50s-60s (typical)	Kour et al. [5]; Pottinger et al. [17]
Initial symptoms	Limb weakness, bulbar symptoms	Limb weakness	Segura et al. [2]
Progression rate	Rapid (loss of ambulation in 2-3 years)	Slower (ambulation loss varies)	Pottinger et al. [17]; Kumbier et al. [18]
Cognitive impairment	Frequent (e.g., executive dysfunction)	Less common (e.g., in C9orf72)	Salah et al. [12]; Pottinger et al. [17]
Respiratory complications	Early and severe	Later onset	Segura et al. [2]
Median survival	Shorter (approximately 2-3 years)	Longer (~3 to >5 years)	Pottinger et al. [17]
Genetic basis	FUS mutations	SOD1, TARDBP, C9orf72	Provasek et al. [15]; Kumbier et al. [18]
Molecular pathophysiology	FUS mislocalization, RNA metabolism disruption	Diverse mechanisms, slower RNA metabolism disruption	Kumbier et al. [18]; Assoni et al. [19]

While ALS8 has slower disease progression, it is marked by aggressive cognitive and behavioral disturbances, such as executive dysfunction, apathy, and anosognosia, which are more prevalent compared to other subtypes (Table 1). This dual burden of physical and cognitive decline significantly complicates management and contributes to a poorer prognosis [17,18]. These unique attributes make ALS8 a critical focus for developing targeted therapies that may also benefit other ALS subtypes. 

Genetic basis of ALS8

ALS8 is primarily associated with mutations in the VAPB gene, particularly the p.P56S mutation, which disrupts cellular membrane trafficking and impairs the ER stress response, contributing to its pathogenesis [20]. This mutation leads to an atypical unfolded protein response (UPR). It is characterized by the upregulation of pro-apoptotic genes such as CHOP and ATF4 [21]. However, there is no corresponding increase in BiP levels, indicating a partial disruption of the UPR. The persistence of altered gene expression in motor neurons, particularly in models derived from induced pluripotent stem cells (iPSCs), further exacerbates neuronal stress and cell death, contributing to the aggressive nature of the disease [15].

Animal models with this mutation showed an early activation of the PERK-mediated stress response [21]. This ultimately led to ATF4/CHOP-driven motor neuron degeneration. This feature distinguishes ALS8 from other subtypes, such as ALS1, which involves SOD1 mutations and a distinct protein misfolding profile [21,22]. In contrast, other studies suggest links between ALS8 and mutations in the valosin-containing protein (VCP) gene [23,24]. These mutations disrupt protein degradation, leading to misfolded protein accumulation and nerve cell damage, a mechanism distinct from oxidative stress caused by SOD1 mutations in ALS1 or RNA processing disruptions in ALS6 [23,24]. 

The development of ALS8 has also been associated with mutations in the FUS gene, which codes for a protein essential to RNA metabolism and the regulation of stress granules [15]. Researchers have identified over 50 different mutations in the FUS gene among ALS patients [15]. These mutations interfere with normal cellular functions, causing the FUS protein to become mislocalized, ultimately leading to neurodegeneration. In contrast, other ALS variants, such as those involving SOD1 or C9orf72 mutations, have unique genetic characteristics and disease pathways, emphasizing the diverse nature of ALS [17].

Collectively, these genetic insights highlight the heterogeneity of ALS, with ALS8 exhibiting a unique molecular profile compared to other subtypes, such as those involving C9orf72 or TARDBP mutations [15,17]. This variability underscores the need for targeted therapies that address the specific genetic pathways affected in ALS8, offering opportunities for broader therapeutic windows due to its slower progression compared to other ALS types [20].

Current pharmacological approaches for ALS8

The management of ALS8 involves the use of several existing ALS treatments, although their effectiveness and outcomes vary. Riluzole, a glutamate antagonist, is the first-line treatment and has been shown to moderately slow disease progression and extend survival in ALS patients, including those with ALS8 [2]. Edaravone, an antioxidant that reduces oxidative stress, offers modest benefits, particularly when initiated early, but its effectiveness in ALS8 remains limited, with reported side effects such as headaches and skin reactions [17,25]. Sodium phenylbutyrate/taurursodiol, a newer therapeutic, shows promise in extending survival and slowing progression, positioning it as a potentially valuable option for ALS8 [26]. However, these treatments focus primarily on symptom management and slowing progression, without halting the disease entirely, emphasizing the need for targeted therapies.

Pharmacological options tailored specifically for ALS8 are currently lacking. Emerging treatments are exploring pathways involved in FUS-mediated neurodegeneration, the hallmark of ALS8, such as enhancing autophagy and mitigating oxidative stress [5]. Experimental approaches, including gene therapy and small-molecule therapies, aim to target these unique molecular mechanisms [27,28]. For example, therapies like tofersen, designed for ALS1 with SOD1 mutations, highlight the potential for subtype-specific drugs that could be adapted for ALS8 [29].

Innovative supportive care strategies are also being investigated. Wearable technologies, such as the hybrid assistive limb (HAL), show potential in improving ambulatory function in patients with neuromuscular diseases and could complement pharmacological treatments for ALS8 [30]. Despite these advancements, the complexity of ALS8's pathophysiology and its variability among patients continue to pose significant challenges. Future research should prioritize the development of targeted, individualized treatment strategies to address both the motor and non-motor symptoms of ALS8.

Emerging therapeutic approaches

Emerging research in ALS8 treatment is exploring precision medicine approaches, including CRISPR/Cas9 gene editing and RNA-based therapies, alongside regenerative approaches like stem cell treatments. CRISPR/Cas9 offers the potential to correct specific mutations in genes implicated in ALS8, such as VAPB or FUS, by restoring normal cellular function and halting neurodegeneration [15,31]. Preclinical studies have shown promise in reversing the accumulation of toxic protein aggregates and restoring RNA metabolism [15].

RNA-based therapies, including antisense oligonucleotides (ASOs) and RNA interference (RNAi), are also being investigated to selectively silence mutated alleles while preserving normal gene function. These therapies have demonstrated success in preclinical models for other ALS subtypes, like SOD1 and C9orf72, and efforts to develop similar approaches for VAPB-related ALS8 are in the early stages [5,32].

In contrast to genetic interventions, stem cell therapies aim to replace damaged motor neurons and promote neuronal repair and regeneration. While they do not directly target the genetic causes of ALS8, their potential to restore functional neural networks makes them a promising adjunctive approach. However, challenges such as delivery methods, off-target effects, and concerns about viability and unintended consequences, including immune reactions or tumorigenicity, must be addressed in ongoing research [17]. By combining genetic interventions like CRISPR/Cas9 and RNA-based therapies with regenerative strategies such as stem cell treatments, researchers aim to develop a comprehensive approach to combat ALS8. Continued research and clinical trials will be essential to overcoming the challenges and translating these experimental therapies into safe and effective treatments for ALS8 patients.

Symptom management and supportive therapies for ALS8

Symptom management in ALS8 requires a multidisciplinary approach tailored to its unique progression, focusing on respiratory, motor, and nutritional support. Respiratory therapies, such as noninvasive ventilation, become critical as respiratory muscle weakness progresses. However, these can be complicated by bulbar dysfunction, which may occur later in the disease [2]. Nutritional support, including gastrostomy feeding, is essential in advanced stages to prevent malnutrition, significant weight loss, and aspiration pneumonia, all of which exacerbate the disease's clinical burden [17].

Physical and occupational therapies play a vital role in maintaining strength, mobility, and independence, aligning with approaches used in other ALS subtypes. However, the slower progression of ALS8 allows for more proactive and sustained symptom management compared to faster-progressing forms, enabling longer periods of mobility and functional independence [7]. Additionally, psychological support is crucial for addressing the emotional burden associated with cognitive decline and disease progression, particularly in ALS8, where cognitive symptoms are more prominent [12]. The integration of these supportive measures into individualized treatment plans optimizes patient outcomes and enhances quality of life. By combining respiratory, nutritional, and motor support with psychological care, clinicians can better address the complex and evolving needs of ALS8 patients.

Challenges and limitations in ALS8 research and treatment

Research and treatment of ALS8 face significant challenges, primarily due to its rarity and limited patient population. The small number of patients makes it difficult to recruit participants for clinical trials, restricting the ability to collect robust data on treatment efficacy and safety [2,24]. Additionally, the complex genetic and phenotypic variability among ALS patients complicates the development of standardized treatment protocols and targeted therapies.

Gaps in understanding the precise mechanisms of ALS8 further hinder progress. While the p.P56S mutation in the VAPB gene and its impact on ER stress responses are well-documented, the detailed molecular pathways leading to neurodegeneration remain poorly understood. This lack of clarity impedes the development of targeted interventions and necessitates further investigation into both pathogenic and protective genetic factors, such as ALKBH3, which may influence disease progression [15,17].

Advanced therapeutic approaches, such as gene editing and RNA-based therapies, also face barriers due to high costs, ethical considerations, and technical complexities [31]. Moreover, while ALS8’s slower progression offers a valuable therapeutic window, this same feature can delay the urgency of interventions, complicating study timelines and patient recruitment. Collaborative efforts across the scientific, medical, and patient communities are essential to overcome these obstacles and advance the development of effective therapies for ALS8.

## Conclusions

ALS8 is a rare and distinct subtype of amyotrophic lateral sclerosis, marked by mutations in the VAPB gene and a slower disease progression. These characteristics present a valuable opportunity for targeted therapeutic interventions, but the rarity of ALS8 and the limited understanding of its molecular pathways pose significant challenges. Current treatments remain largely palliative, underscoring the urgent need for research into novel approaches such as gene editing, RNA-based therapies, and stem cell treatments to address its underlying mechanisms.

Integrating innovative treatments with robust symptom management and supportive care can improve the quality of life and functional independence of ALS8 patients. This review highlights the critical role of precision medicine and multidisciplinary care in meeting the unmet needs of ALS8. Advancing research on its pathophysiology and genetic drivers could not only improve outcomes for ALS8 patients but also provide insights that benefit the broader ALS community.
